# Cortical visual processing evokes short-latency reward-predicting cue responses in primate midbrain dopamine neurons

**DOI:** 10.1038/s41598-018-33335-9

**Published:** 2018-10-08

**Authors:** Norihiro Takakuwa, Peter Redgrave, Tadashi Isa

**Affiliations:** 1 Department Dev. Physiol., Nat’l Inst. Physiol. Sci., Okazaki, 444-0864 Japan; 2 Department Physiol. Sci., SOKENDAI, Hayama, 240-0115 Japan; 3 Department Neuroscience, Grad. Sch. Med., Kyoto University, Kyoto, 606-8501 Japan; 4 Department Psychol., University of Sheffield, Sheffield, S10 2TP United Kingdom

## Abstract

After classical conditioning dopamine (DA) neurons exhibit short latency responses to reward-predicting visual cues. At least two possible projections could induce such DA responses; the cortical and subcortical visual pathways. Our recent study has shown that after a lesion of the striate cortex (V1), the superior colliculus (SC), a critical node of the subcortical visual pathway, can mediate short latency cue responses in the DA neurons of macaque monkeys. An obvious question then is does the cortical pathway have a similar capacity? Using the monkeys with a unilateral V1 lesion that took part in the preceding study, we recorded DA activity while they were performing the same classical conditioning task. However, in this study conditioned visual stimuli were presented to the intact visual field, and the effects of ipsilateral SC inactivation were examined. We found that after the SC was inactivated by injections of muscimol both conditioned behavioral responding and reward-predicting, short latency (~100 ms) cue-elicited DA neuronal responses were unaffected These results indicate that the intact cortical visual pathway can also mediate short latency cue elicited responses in DA neurons in the absence of a normally functioning subcortical visual system.

## Introduction

In Pavlovian (classical) conditioning, learned associations between sensory stimuli and subsequent rewards or punishments enable animals to engage appropriate anticipatory conditioned responses^1^. This paradigm has been used widely to investigate errors in reward prediction by the ventral midbrain dopamine (DA) neurons located in substantia nigra pars compacta (SNc) and ventral tegmental area (VTA)^2–4^. Reward prediction errors are the difference between the values of reward predicted by sensory cues and the value that is actually received. Sensory evoked phasic increases in the firing of DA neurones indicate positive reward errors (actual > predicted), while phasic decreases denote negative errors (actual < predicted)^5^. The reward prediction errors signaled by DA neurons are considered critical teaching signals for associative learning, including Pavlovian conditioning^6,7^. While reward prediction error responses are widely considered an essential component of DA signaling, the sources of afferent sensory signals that represent actual and predicted reward values remain unclear^8^. Since much of the work investigating reward prediction error signaling by DA neurons in primates has used visual conditioned stimuli^5,9,10^, we directed our attention in this study to investigate further the visual sensory processing providing input to DA neurons.

In primates the cortical visual system expanded to enable the analysis of complex visual features including high-spatial frequency form, colour and texture that comprise every-day visual scenes^11,12^. However, throughout much of vertebrate brain evolution most species have relied on a conserved phylogenetically ancient subcortical visual system involving direct retinal projections to the optic tectum/superior colliculus^13,14^. This system is specialised for the detection of relatively simple visual features associated with local luminance changes^15^. Recently, the existence of a direct pathway from the superior colliculus (SC), a critical node in subcortical visual processing, to DA neurons in the ventral midbrain has been demonstrated in a range of mammalian, species including primates^16^, rodents^17^ and carnivoras^18^. This suggests the tecto-nigral projection is a conserved feature of mammalian brain organization, and provides a direct route whereby visual conditioned stimuli can induce short-latency phasic visual responses in ventral midbrain DA neurones^19^. This was confirmed in our previous study^10^, in which a unilateral lesion of striate visual cortex (V1) ensured that only subcortical and extrastriate cortical visual processing was functional on one side of the brain^20,21^. With this preparation we found that behavioural Pavlovian conditioning was preserved when visual conditioned stimuli were presented in the V1 lesion-affected visual field. A critical role for the SC was established by showing that pharmacological inactivation of the SC ipsilateral to the V1 lesion suppressed normal visually-evoked classically conditioned responding. To investigate further the role of the SC in visual Pavlovian conditioning, we examined the phasic responses of putative ventral midbrain DA neurons evoked by the behaviourally effective conditioned visual stimuli^10^. Reliable short latency (~100 ms) value-coded DA responses were evoked by conditioned visual stimuli presented at different locations within the V1 lesion-affected visual field. These neural responses were also blocked by local pharmacological inactivation of the ipsilesional SC. Together our previous results demonstrated that subcortical visual processing involving the SC has the capacity to support visual Pavlovian conditioning and the short-latency phasic activation of ventral midbrain DA neurons, independent of any cortical involvement, including non-striate visual cortex.

However, our previous study^10^ begs the question of whether the evolutionary more recent striate visual cortex also has the ability to support visual Pavlovian conditioning and evoke short latency phasic activation of DA neurons, independently of the long established subcortical visual processing in the SC. To address this issue, we used the same unilaterally V1 lesioned primate preparation^10^, except visual conditioned stimuli were presented to the intact cortical visual system, i.e. the visual field contralateral to the intact V1. To test the possible exclusive role of cortical visual processing, both visually conditioned behaviour and the activity of contralateral DA neurons were recorded before and after the contralateral SC was pharmacologically inactivated. In this design the properties of cortical visual processing could be observed independently of concomitant input to DA neurons from the SC.

## Results

### Pavlovian conditioning task

**Figure 1:**
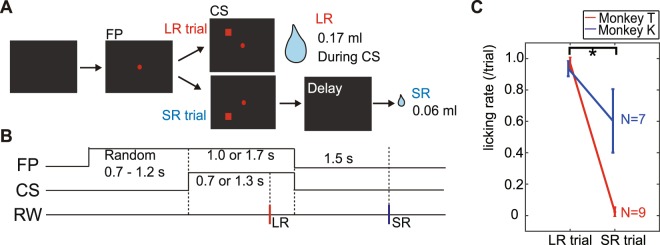
Pavlovian conditioning task. (**A**,**B**) The Pavlovian conditioning task used in this study. Monkeys were required to acquire a central fixation point (FP) after which visual CS was presented their left or right visual field (intact visual field ipsilateral to the V1 lesion). Anticipatory licking that occurred after CS onset and reward delivery was recorded as a measure of conditioned responding. Abbreviation; FP (fixation point), LR (large reward), SR (small reward), CS (conditioned stimulus). (**C**) Averaged licking rate during CS presentation in LR and SR trials is plotted for both monkeys. There is significant difference between LR and SR trials (Wilcoxon signed-rank test, α < 0.05).

In this study, monkeys performed a simple Pavlovian visual conditioning task shown in Fig. 1A,B. Monkeys were required to maintain their gaze on a central fixation point (FP) throughout the trial. This was to ensure that subsequent conditioned visual stimuli were directed to known locations within the animal’s visual field. The next step was to present conditioned stimuli (CSs) to the visual field contralateral to the intact visual cortex, (i.e. the visual field ipsilateral to the V1-lesion). Two conditioned stimuli were used, one predicting a large reward (LR CS – delivered within the CS period) and another predicting a small reward (SR CS – delivered after CS offset). The only means of discriminating the conditioned stimuli was the location where they were presented within the visual field (above and below the FP). During the electrophysiological recording sessions, monkeys showed CS-evoked conditioned anticipatory licking responses (Fig. 1C; Wilcoxon signed-rank test, P = 0.0039, N = 9 in monkey T and P = 0.0156, N = 7 in monkey K). Licking rate during the period between CS onset and LR delivery, was significantly higher in LR trials than that in SR trials (when the SR was delivered after SC offset). These conditioned responses (CRs) showed that the monkeys could effectively discriminate the visual CSs that predicted different amounts of subsequent reward. Monkeys were well practiced on this Pavlovian conditioning task having had experience of it for more than one year before the current experiments.

### Conditioned responses after the SC inactivation

**Figure 2:**
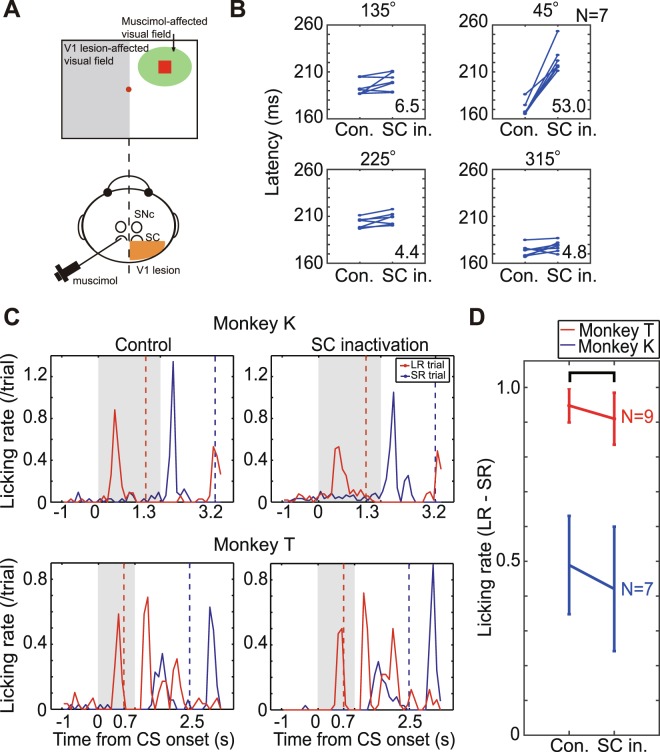
Conditioned responses after the SC inactivation. (**A**) Schema of the experimental design to determine the effects of inactivating the SC. Visual CSs were presented to the visual field contralateral to the intact V1, and muscimol was injected into SC ipsilateral to the intact V1. (**B**) Latencies of visually guided saccades from the target onset. The latencies of saccades to the muscimol-affected visual field (45°) were prolonged during SC inactivation, while saccades to the other target locations were unaffected. Numerals in individual plots indicate difference of latencies (in ms) between before and after the muscimol injection. (**C**) Typical examples of licking behavior before (left two panels) and during the SC inactivation (right two panels) in each monkey (monkey K; upper two panels, monkey T; lower two panels). Gray hatch areas indicate the period of CS presentation, dashed lines indicate time of reward delivery in LR (red) and SR (blue) trials. (**D**) Average of difference in licking rate between LR and SR trials. There was no significant difference between before and during the SC inactivation in both monkeys (Wilcoxon signed-rank test, α < 0.05).

To address the question of whether visual information processed by the cortical pathway can support visual Pavlovian conditioning, independently of subcortical visual input, we blocked neural activity in the SC with microinjections of the GABA agonist muscimol at locations within the collicular map where the LR-CS would be represented (Fig. 2A). The appropriate location for the SC inactivation was confirmed by prolongation of saccadic latencies in visually guided saccade task^22,23^ (Fig. 2B). The latencies of saccades to muscimol-affected visual field (45°) were clearly prolonged during the SC inactivation (53 ms longer after the inactivation; two-sample t-test, P < 0.00001), while saccades to other target locations were unaffected.

In the visual Pavlovian conditioning experiments conditioned licking was observed before and after inactivation of the appropriate location in the SC (Fig. 2C). In the pre-injection control trials, conditioned anticipatory licking was observed in the period between CS onset and RW delivery in LR trials, and after CS offset in the SR trials (left two panels in Fig. 2C). After inactivation of the SC, the conditioned responding was not significantly altered. Anticipatory licking was observed at different times before the delivery of both large and small rewards (right two panels in Fig. 2C). In the period between CS onset and the time of large reward delivery the anticipatory licking between LR trials and the SR trials was not reliably different in trials conducted before and after SC inactivation (Fig. 2D, (Wilcoxon signed-rank test, P = 0.1289, N = 9 in monkey T (red) and P = 0.2969, N = 7 in monkey K (blue)). Furthermore, we adopted a boot-strapping permutation test to evaluate the observed licking data with multiple iterations of randomized data assignment. We were unable to find any reliable difference in the observed licking rate before and during the SC inactivation compared with randomly shuffled data (Table 1 in the Supplementary information). These results indicate that cortical visual processing can support classically conditioned behavioural responding when subcortical visual processing at spatially corresponding locations within the SC had been suppressed.

### DA responses after the SC inactivation

**Figure 3:**
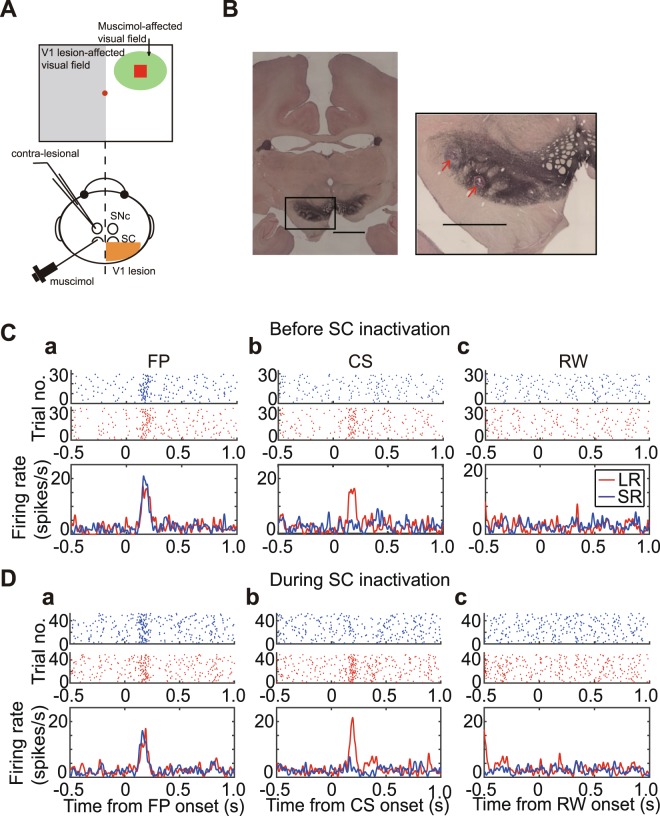
DA responses after the SC inactivation. (**A**) Schema of the experiments with single unit recording from midbrain DA neurons. The unit activity was recorded from the SNc or VTA ipsilateral to the intact V1. (**B**) Recording sites marked by electrolytic lesions in a brain section stained with anti-TH immunohistochemistry. The lesions were located among TH-immuno-stained neurons. Scale bar; 5 mm in left panel, 3 mm in right panel. (**C**,**D**) A typical DA responses before (**C**) and during (**D**) the SC inactivation. Raster plots were sorted on the top of figures in LR (red) and SR trials (blue), receptively. These figures are aligned to the onsets of FP (a), CS (b) and RW (c), respectively.

Insofar as cortical visual processing is sufficient to support value-dependent Pavlovian conditioned responding evoked by visual CSs, it is pertinent to ask if the results of cortical visual processing of CSs is also sufficient to elicit value-coded short-latency phasic responses in DA neurons when subcortical visual processing in the SC is blocked. To address this question, we performed single unit recordings from putative midbrain DA neurons ipsilateral to the intact V1 (Fig. 3A,B) during the Pavlovian task. Traditional criteria were used to classify the 9 putative DA neurons we succeeded recording continuously throughout the period before and after SC inactivation. (see Methods and Takakuwa *et al*.^10^).

#### DA Response Magnitudes

Initially, DA activity evoked by visual CSs in the Pavlovian paradigm was recorded before inactivating the SC with an injection of muscimol (Pre-inactivation block = ~60 trials). During these trials prominent short-latency phasic responses were reliably elicited by the onset of the fixation point (Fig. 3Ca, Supplementary Fig. 3Aa). This behaviorally relevant event indicated to the monkey that a trial was about to start. The amplitude of this DA response was not different between LR and SR trials (Fig. 3Ca, Supplementary Fig. 3Aa), presumably because at this point within the trial the monkeys were unsure of the value of future reward that would be predicted by the CS. In contrast, the succeeding LR and SR CSs evoked putative DA responses that reflected the different values of predicted rewards (Fig. 3Cb); significant responses were reliably evoked by the LR CS, while responses elicited by the lower value SR CS were weak or absent (Supplementary Fig. 3Ab). Finally, delivery of the predicted rewards failed to cause any notable changes in the baseline activity of the putative DA neurons, whose activities were not different between LR and SR trials (Fig. 3Cc, Supplementary Fig. 3Ac). After inactivation of the SC, activity patterns of DA neurons were not different from those recorded before the inactivation. Larger responses were elicited when the LR CS was presented than those evoked by the SR CS (Fig. 3Db and Supplementary Fig. 3Db). Similarly, there were no significant differences in responses to the FP and to RW presentation between in LR and in SR trials (Fig. 3Dac and Supplementary Fig. 3Dac).

**Figure 4:**
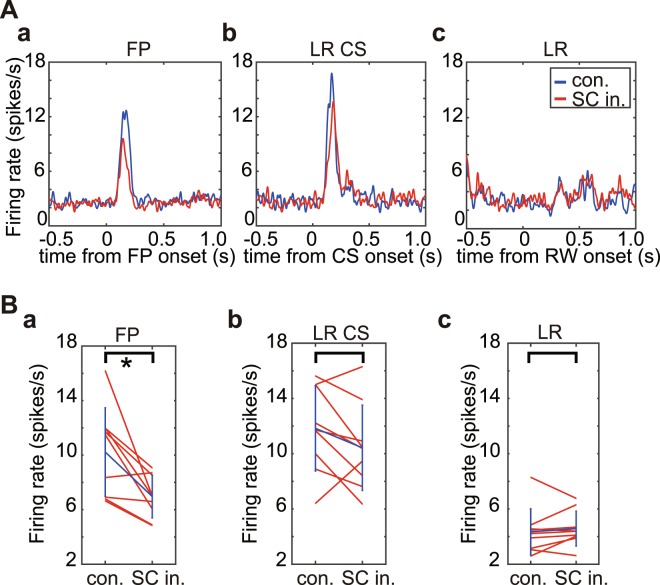
Comparison of DA responses before and after the SC inactivation. (**A**) Averaged spike density functions of DA responses before (blue lines) and during (red lines) SC inactivation. These figures are aligned to the onsets of FP (a), LR CS (b) and LR (c), respectively. (**B**) Firing rates of individual DA neurons. The firing rate was calculated within the time windows (100–300 ms from FP (a) and LR CS (b), 150–350 ms from LR (c)). The averaged scores and SDs of the firing rate before (con.) and during SC inactivation (SC in.) are indicated in blue lines. *Significant difference (Wilcoxon signed-ranks test, α < 0.05).

An analysis of DA response data at the population level is illustrated in Fig. 4. Averaged spike density functions (Fig. 4A), and peak firing rate of each of the 9 recorded DA neurons (Fig. 4B), before (blue trace) and after the SC inactivation (red trace) are shown for responses evoked by the FP, the LR CS, and the LR. At the population level, firing rate responses to the FP (100–300 ms from FP onset) were slightly, but significantly reduced after the SC inactivation (Fig. 4Aa,Ba, Wilcoxon signed-ranks test, α < 0.05, N = 9, p = 0.0117 (FP), p = 0.1289 (LR CS), p = 0.4258 (LR)). However, responses to CS (Fig. 4Ab) and RW (Fig. 4Ac) before and after the SC inactivation were not reliably different. Analysis of data from individual neurons (Fig. 4B) indicated that SC inactivation caused the firing rate of 7/9 neurons to decrease, 4 significantly so (two-sample t-test, α < 0.05). While for 2/9 DA neurons SC inactivation increased their rate of phasic firing, one significantly so (two-sample t-test, α < 0.05). Furthermore, we also adopted the permutation test to the DA responses to LR-CS before and during SC inactivation (Table 2 in the Supplementary information). The firing rate of the same 2/9 neurons was significantly decreased, while for the remaining 7/9 neurons, inactivating the SC had no statistically reliable effect.

In summary, after inactivating the SC location where the LR-CS would be represented, the phasic responses of 9 putative-DA neurons to each of the various stimuli in the Pavlovian paradigm in terms of average response magnitudes was essentially unaltered (Fig. 3Da–c - Supplementary Fig. 3Ba–c). These results indicate that cortical visual processing was sufficient to elicit similar magnitude short-latency phasic responses in ventral midbrain DA neurons, independently of visual processing at spatially corresponding locations within the SC.

#### DA Response Latencies

**Figure 5:**
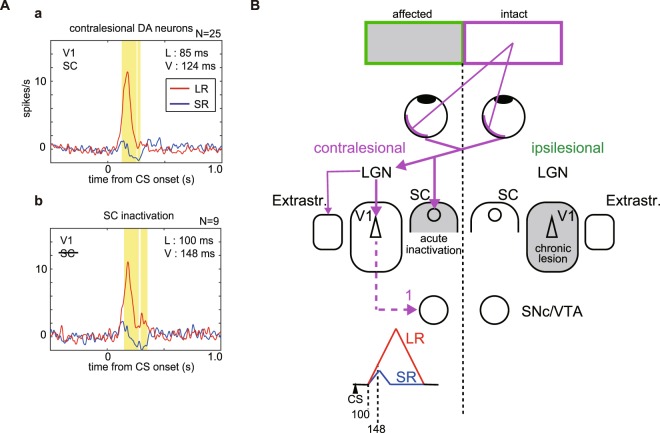
Visual input pathways and onsets of DA responses to visual CSs. (**A**) Averaged spike density functions of DA responses to CSs recorded on the contralesional side before and during SC inactivation (before inactivation; panel a, during inactivation; panel b). The earliest time point when the activity exceeds 2 SD from the baseline activity (−500–0 ms) are indicated in the panels as latencies (L). The earliest time point when value differentiation emerged between LR and SR trials are indicated as value differentiation (V). Yellow areas in the figures show the period during which the responses to LR-CS and SR-CS trials were significantly different for more than 15 ms bin (N = 25 in a, N = 9 in b, two-sided sign test, a <0.05). Left top letters (V1 and SC) indicate available visual input pathways. (**B**) A schematic diagram indicating the afferent visual pathways responsible for evoking value-coded DA responses to classically conditioned visual stimuli. The chronic lesion of the unilateral V1 and acute inactivation of the SC limit the possible visual input pathways to the cortical visual pathway (purple continuous and dotted lines through the LGN and V1, “1”).

One way in which DA responses evoked by cortical and subcortical visual processing might be expected to differ would be in terms of response latencies. Insofar as there is a relatively direct subcortical retino-tecto-nigral route to DA neurons^16,17^, the way in which cortical visual processing can trigger a phasic DA response is likely to be more indirect, and therefore possibly take longer. Therefore, to compare DA response latencies evoked by CS onset in the intact visual field before and after SC inactivation we used two criteria for latency measurements. One was the earliest time point when the averaged spike density function of DA responses to LR CS exceeded 2SD above the baseline (‘L’ in Fig. 5A). The second was the earliest time points when value differentiation emerged between LR and SR trials, indicated as value differentiation calculated by the two-sided sign test (a < 0.05; ‘V’ in Fig. 5A). When the LR CS and SR CS were presented in the visual field of the intact V1 cortex prior to the SC inactivation the’L’ and ‘V’ values of the DA response latencies were 85 ms and 124 ms, respectively (Fig. 5Aa). After SC inactivation the ‘L’ and ‘V’ DA response latencies DA were proportionally longer (‘L’ = 100 ms and ‘V’ = 148 ms,) (Fig. 5Ab), but not reliably so. These results suggest that, despite a potentially longer route to the ventral midbrain, cortical processing of visual CSs can trigger phasic DA responses with short latencies comparable to those evoked by sub-cortical visual processing^10^.

## Discussion

The purpose of the present study was to test whether cortical visual processing can support visual Pavlovian conditioning and evoke short-latency phasic DA responses independently of contribution by the midbrain SC. To evaluate the role of cortical visual processing we used monkeys with unilateral V1 lesions. Here we presented conditioned visual cues in the visual field contralateral to the intact V1 before and after suppression of neural activity in the ipsilateral SC. The main findings were that both the classically conditioned anticipatory behavioural responses and the short-latency phasic responses of putative DA neurons were largely unaffected by inactivation of the SC. Ideally, the same procedures for inactivating the V1 and SC would have been used, however, because the V1 is a far larger structure than the SC it would have be impractical to inhibit it completely with muscimol, as in the case of SC. However, our results safely suggested that the cortical visual processing can provide comparable input to the neural mechanisms responsible for Pavlovian conditioned responding, and to evoke similar value-coded short-latency phasic responding in ventral midbrain DA neurons.

Without knowing how visual information via the cortical and subcortical pathways together or separately influence DA activity, it is difficult to predict how information from the two sources might combine. Here, we succeeded recording DA activity from 9 neurons continuously throughout the trials before and during the SC inactivation. We revealed that DA responses can be elicited by visual information via the cortical pathways, and there was tendency for the magnitude of the LR CS responses to decrease and ‘L’ and ‘V’ latency values to become longer after SC inactivation. However, if there were differences in the magnitude or latency of DA responses before and after the SC inactivation, they were small and beyond our ability to detect them. Thus at the population level, we found no significant differences in firing rate of DA responses to LR CS before and after SC suppression (Fig. 4Bb), despite the response magnitudes of 4 individual neurons being significantly decreased and 1 neuron significantly increased by SC inactivation (two-sample t-test, α < 0.05). This suggests that with a larger sample, statistically significant differences might become apparent at the population level. Further, when comparing the magnitude of DA responses to visual CSs mediated via the cortical pathway (in this study; 10.43 ± 3.09 spikes/sec) and the subcortical pathway (Fig. 3H in Takakuwa *et al*., 2017; 12.42 ± 3.82 spikes/sec) (two sample t-test, P = 0.1539), no statistically reliable difference was found. Similarly, the magnitude of the DA evoked by the presentation of the LR-CS to the intact (12.40 ± 3.61 spikes/sec, Fig. 4B) and lesion-affected (12.42 ± 3.82 spikes/sec, Fig. 3H in Takakuwa *et al*., 2017) visual field (two sample t-test, P = 0.9781) were also not reliably different. Together, these results suggest that inputs to the ventral midbrain from the cortical and subcortical visual systems can operate independently, and produce comparable reward-predicting DA responses.

We also compared the latencies of DA responses observed when CSs were presented to the V1 lesion-affected visual field (taken from Takakuwa *et al*., 2017; Supplementary Fig. 5A) with those recorded with intact V1 in the present study. We found that the average ‘L’ and ‘V’ latencies were 7 ms and 26 ms shorter than the corresponding values obtained when the LR-CS was presented to the intact cortical visual field. Again these differences were not statistically significant. Thus, the L’ and ‘V’ values of DA responses on the ipsilesional side that were elicited by CSs in intact visual field (Supplementary Fig. 6Aa and the purple lines (“3”) in Supplementary Fig. 6B) were 93 ms and 112 ms, respectively. While the L’ and ‘V’ values of DA responses on the contralesional side that were elicited by CSs in the affected visual field (Supplementary Figure 6Ab and the green lines (“4”) in Supplementary Fig. 6B) were 92 ms and 110 ms, respectively. These results confirm that visual inputs to the ventral midbrain from V1 and the SC can operate independently, and produce comparable short-latency phasic DA responses. While the tecto-nigral pathway is the likely source of afferent visual input from the SC^16,17,19^, the route by which V1 communicates short-latency visual information to DA neurons in the ventral midbrain remains unknown.

The effectiveness of the SC inactivation was the key to assessing the contribution of the cortical pathway in triggering short-latency visual responses in DA neurons. To this end we made sure that the monkeys performed a visually guided saccadic eye movement task before, during and after the muscimol inactivation. When the appropriate location in the SC was inactivated, the monkeys could still make visually guided saccades, however, the latencies of the saccades only to targets in the muscimol-affected visual field were clearly prolonged (33–201 ms c.f. Aizawa and Wurtz., 1998; McPeek and Keller, 2004).

In our previous study^10^ we used the same procedures for muscimol inactivation of the SC ipsilateral to the unilateral V1 lesion to block contralaterally directed visually guided saccades, to impair visually evoked classically conditioned responses, and completely suppress cue-elicited DA responses on the ipsilesional side. Because all the conditioned cue-elicited DA responses disappeared after SC inactivation in animals with chronic V1 lesion we can conclude that visual projections from the lateral geniculate nucleus to extrastriate cortex play no role in eliciting visually evoked short-latency phasic responses in DA neurons in the present Pavlovian task. Thus, taken together with the results of the present paper, we can state that that short-latency visual input to ventral midbrain DA neurons comes, potentially independently, from V1 and/or the SC.

In our previous studies of the “blindsight” phenomenon, we suggested that substantial plasticity occurs after damage to the primate V1^20,24,25^. The present and the preceding study^10^ suggest that plastic changes may have occurred in the SC-mediated pathway to increase the magnitude of the short latency visual cue-evoked responses in DA neurons after the chronic unilateral V1 lesion. This may in part be responsible for the finding that the magnitude of cue-evoked DA responses were not reliably different when the V1 alone was intact (present study), the SC alone was intact^10^, and when both were intact (present study).

A further point is that the present study demonstrated that the cortical visual pathway can also mediate short latency (~100 ms) reward-predicting responses in DA neurons. The fact that we were unable to demonstrate a reliable latency difference when V1 (present study) and the SC were operating alone^10^ was perhaps surprising. Insofar as transmission in the unknown, but necessarily indirect route from V1 to the ventral midbrain might be expected to take longer than transmission in the more direct retino-tecto-nigral route^16,17,20^. Thus, while short latency visual responses (<100 ms) have been recorded in a variety of visual cortical areas (40–100 ms in V1, 50–70 ms in V2, 50–80 ms in V4 and 80–100 ms even in anterior inferior temporal cortex)^26,27^ it is not clear by which route(s) information from these structures is directed to the ventral midbrain (Supplementary Fig. 6). However, we can conclude that when simple visual cues are used (luminance change at different locations), cortical and subcortical visual processing can trigger similar magnitude DA responses with comparably short latencies.

## Methods

### Animals

We used two adult Japanese monkeys (Macaca fuscata; both female, body weight 5–7 kg, monkeys T and K). A head holder was implanted in each monkey, and the monkey’s head position was fixed during each experiment. The V1 in each monkey was unilaterally lesioned before training for the present classical conditioning task^10^. All procedures were performed in accordance with the National Institutes of Health Guidelines for the Care and Use of Laboratory Animals and approved by the Committee for Animal Experiment at the National Institute of Natural Sciences.

### Surgery

All the surgeries were performed under isoflurane anesthesia (1.0–1.5%) (see Yoshida *et al*.^20^). The left V1 of monkey T, and the right V1 of monkey K were surgically removed by aspiration. The opercular surface of the striate cortex and medial area in the Calcarine Sulcus were removed, while the ventrolateral part of the opercular surface, which encodes foveal vision (visual field for eccentricity 0 to 1.0°) remained intact (Supplementary Fig. 1).

### Behavioral task

We used a real-time experimental control system (Tempo for Windows, Reflective Computing; http://reflectivecomputing.com/) for visual stimulus presentation and data collection. A monitor (Diamondcrysta WIDE RDT272WX (BK), MITSUBISHI) was positioned 34.5 cm in front of the monkeys’ eyes. Eye movements were measured with a video-based eye tracker (EYE-TRAC 6; Applied Science Laboratories, sampling rate: 240 Hz).

Our classical conditioning task sequence was described in detail in our previous report (Takakuwa *et al*., 2017). Briefly, conditioned stimulus (CS) (2.2° red square, luminance contrast: Michelson contrast 0.87 (Weber contrast 13.4) against the background of 1.0 cd/m^2^) was presented in either the upper (eccentricity: 10°, direction: 45° relative to the horizontal axis from central fixation point; FP) or lower quadrant (eccentricity: 10°, direction: −45° relative to the horizontal axis from the central FP) of the intact visual hemifield. Monkeys were required to maintain fixation during FP presentation. If their gaze deviated from FP (size, approximately 2.5° radius), the trial was terminated immediately. A CS appeared from 0.7 to 1.2 s after FP onset and was presented for 1.0 s (monkey T) or for 1.7 s (monkey K). Two CSs, one predicting a large reward (LR CS) and the other a small reward (SR CS) were randomly assigned. The CSs could be discriminated by their positions from central FP. Assignment of the two CSs were maintained throughout a daily session. The reward spout was placed in front of monkey’s mouth and had to be licked to obtain juice reward. Bouts of licking were recorded by a photo-detector, and an individual lick was counted when the monkeys’ tongue approached the reward spout. To evaluate the acquisition of a conditioned licking response (CR), the number of licks in 0.1 s time bins between CS onset and reward delivery (0.7–1.3 s after CS onset) was used to determine the licking rate measure.

### Muscimol injections

To evaluate the contribution of V1 to visual Pavlovian conditioning, we recorded anticipatory conditioned responding (licking) in the period before and during inactivation of the SC. The inactivation was induced by an intracollicular microinjection of the gamma aminobutyric acid A (GABA_A_) receptor agonist, muscimol. The injection site was determined by a preceding electrophysiological test which identified the location of neurones within the SC’s retinotopic map that were responsive to LR CS onset. Muscimol (concentration: 1.0 μg/μL, the total volume: 0.5 μL) was pressure-injected (0.4 μL/min) through a 27–gauge needle connected to a 10 μL Hamilton syringe (Hamilton Company, Reno, Nevada, USA) mounted on a syringe pump.

Each experimental session consisted of a control block of about 60 trials which established a baseline for conditioned responding. When completed, muscimol was then injected into the SC. The inactivation block started from 0–20 min after the muscimol injection and continued for at least1 hour.

### Recording from DA neurons

Single unit recordings from putative DA neurons in the ventral midbrain were conducted throughout the control and SC inactivation trials. The activity of midbrain DA neurons was recorded with epoxylite-coated tungsten microelectrode (impedance: 9–10 MΩ at 1 kHz, FHC)^10^. In brief, signals were bandpass filtered between 0.1 (or 0.3) and 10 kHz. To identify putative DA neurons, the following criteria were used: (i) On the basis of previously acquired structural MR images the recording location was estimated to be in SNc or the VTA. (ii) the presentation of an unpredicted reward caused a short-latency phasic response. (iii) neurons exhibited low baseline activity (1.0–10.0 Hz)^9,28^. (iv) a spike width that was clearly longer than those of nearby SNr neurons that had baseline firing rates > 40 Hz^28–30^.

### Histology

Two small electrolytic lesions were made in each recording track (20 µA, 30 s) to confirm recording sites in monkey K. Coronal sections (40 µm) of the tissue that included SNc were immunostained for tyrosine hydroxylase (TH) to reveal the location of DA neurons (Fig. 3B).

## Electronic supplementary material

Supplementary information
