# Supplementary material for: Cortical visual processing evokes short-latency reward-predicting cue responses in primate midbrain dopamine neurons
Source: Sci Rep. 2018 Oct 8;8:14984. doi: 10.1038/s41598-018-33335-9 (PMC6175936; doi:10.1038/s41598-018-33335-9)
Supplement: Supplementary file 1 — Supplementary information [file 41598_2018_33335_MOESM1_ESM.pdf]

Supplementary information

Cortical visual processing evokes short-latency reward-predicting cue responses in primate midbrain dopamine neurons

Norihiro Takakuwa[1,2,3], Peter Redgrave[4], Tadashi Isa[1,2,3]

Supplementary figure 1. Unilateral V1 lesion

A The V1 is indicated as red color on the horizontal MRI sections of Monkey K. **B** Lesion areas were indicated as gray area on horizontal MRI sections of the Monkeys T and K taken. **C** Thresholds for detecting luminance contrast (Michelson contrast) are distributed at visual field corresponding to the lesion in each monkey. These data were the same as those in our previous paper (Takakuwa et al., 2017).

Supplementary figure 2. Criteria of our putative DA neurons

Spike widths and baseline firing rates of all the recorded neurons were displayed, putative DA neurons (red) and non DA neurons (blue). Spike width was measured as the time between the first negative peak and subsequent positive peak. Our putative DA neuron had long spike waveform (>0.45ms) and low frequency spontaneous activity (0 <, <10 spikes/sec).

Supplementary figure 3. Comparison of firing rates between LR and SR trials before and after the SC inactivation

Magnitude of the DA responses are compared between LR and SR trials before and during the SC inactivation, respectively. The Firing rates were calculated from spike numbers in

the time windows (FP and CS; 100-300 ms from each onset, RW; 150-350 ms from the onset). **A** DA responses to FP (a), CS(b) and RW(c) before the inactivation were shown as control results. There was no significant difference in responses to FP and RW. On the other hands, the firing rate to CSs were significant different between LR and SR trials (Wilcoxon signed-ranks test, $\alpha < 0.05$, N=9, p=0.847 (FP), $p < 0.001$ (CS), p= 0.437 (RW)). **B** DA responses during the inactivation were shown. There was no significant difference in responses to FP and RW, but the firing rate to CSs were significant different between LR and SR trials (Wilcoxon signed-ranks test, $\alpha < 0.05$, N=9, p=0.288 (FP), $p < 0.001$ (CS), p= 0.119 (RW)).

Supplementary figure 4. DA responses in SR trials during the SC inactivation

A Averaged DA responses to SR CS (left) and SR (right) before (blue line; con.) and during (red line; SC in.) the SC inactivation. **B** Firing rates of individual DA neurons. The firing rate was circulated with time windows (100 - 300 ms from SR CS (left) or 150 - 350 ms from SR (right)) before (con.) during the SC inactivation (SC in.). Average values and SD are indicated in blue lines. There was no significant difference between control and SC inactivation. (Wilcoxon signed-ranks test, $\alpha < 0.05$, N=9, p=0.25 (SR CS), p= 0.625 (SR))

Supplementary figure 5. Visual input pathways and onsets of the DA response to visual CSs mediated by the SC.

Ipsilesional side version of Figure 5. **A** Averaged spike density functions of DA responses to CSs recorded on the ipsilesional side before and during SC inactivation (before; panel a, during; panel b). These data were taken from our previous paper (Takakuwa et al., 2017). **B** A schematic diagram indicating the visual input pathways (green continuous and dotted lines) to DA responses, mediated by the SC, on the ipsilesional side after the chronic lesion of V1.

Supplementary figure 6. Visual input pathways and onsets of the DA response to visual CSs mediated by both SC and V1.

A DA neurons were recorded from ipsilesional (a) and contralesional (b), respectively. **B** Schematic visual input pathways for DA responses in this condition. CSs were presented into intact visual field in case of (**Aa**) and in the affected visual field in case of (**Ab**). In the case of (**Aa**), visual information was mediated by both via the SC (extrageniculate) and in the V1 (geniculostriate) on the contralesional side (purple line "3" in panel **B**). The data on ipsilesional side were taken from our previous paper (Takakuwa et al., 2017). In

the case of (**Ab**), visual information from the affected field was mediated by the SC (extrageniculate; green line “4” in panel **B**).

Table 1. Permutation test on the licking rate between before and during the SC inactivation

To evaluate further any effect of inactivating the SC on conditioned responding, we examined the statistical significance of the difference of licking rate between before and during the SC inactivation by evaluating the 95% confidence interval of the difference (obtained from a permutation test with 5000 resampling). The difference was statistically significant if the 95% confidence internal excluded zero. At first, we calculated that difference of licking rate between LR trials and SR trials (/trial) either before and during inactivation (diff_org, time window; 0 – 0.7 s after CS onset). Second, we shuffled number of licks in each trial including before and during the inactivation within LR trials and SR trials, respectively. We calculated the licking rate again within the first group in which trial number was the same as that before inactivation preceding the shuffling, and the latter group in LR and SR trials, respectively. Then we acquired that difference of licking rate between LR trials and SR trials either before and during inactivation after the shuffling. We repeated the shuffling 5000 times, and investigated whether the original

difference was included in 0.05/N quantile or not. We calculated the upper and lower confidence interval (CI up/low) as (original difference of licking rate – upper/lower quantile of the 5000 differences after the shuffling). We adopted this analysis to all experimental days (Monkey T; 9 days, Monkey K; 7days). In all sessions, upper CI is above 0 and lower CI is under 0. These results suggested that licking patterns were not significantly different between before and during the inactivation.

Table 2. Permutation test on firing rate of DA responses between before and during the SC inactivation

To evaluate further any effect of inactivating the SC on DA responding, we adopted the 5000 times permutation test to DA responses to LR-CS, SR-CS, FP and RW, respectively. We used the time windows for the calculation (CS and FP; 100 – 300 ms from the onset, RW; 150 – 350 ms from the onset).

Regarding the responses to LR-CS, two neurons significantly degreased their firing rate after the SC inactivation, however, the remaining seven neurons did not show significant difference between before and during the SC inactivation. There was significant decrease in their firing rate in responses to FP in 5 neurons, and significant increase in responses to SR in 1 neuron. There was no significant difference in responses to SR-CS and to LR.

Supplementary Information

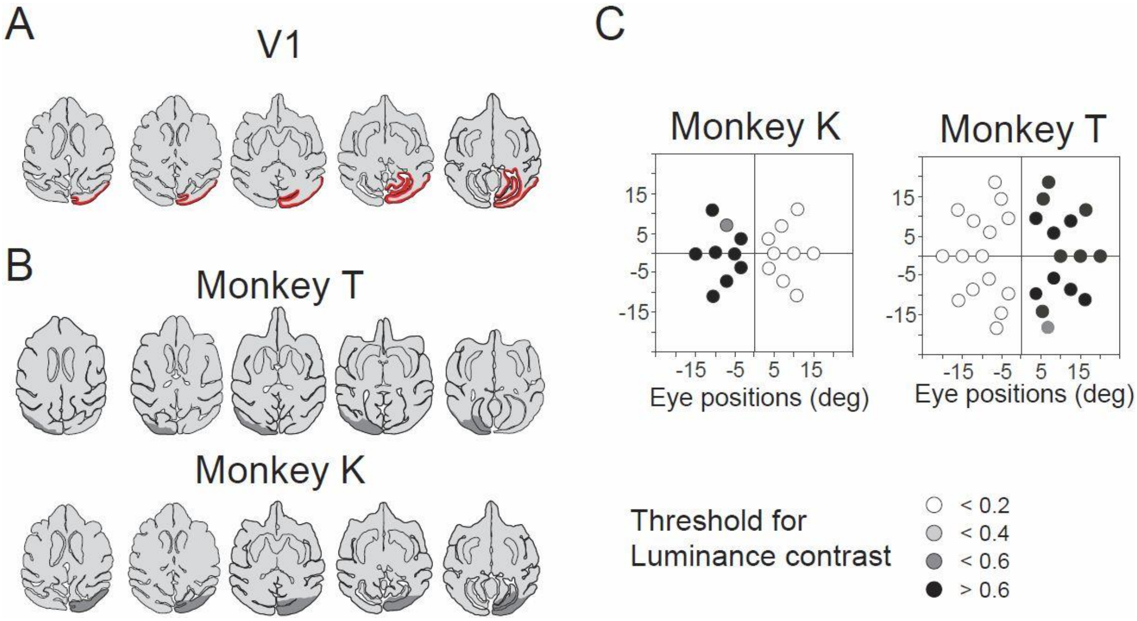

Supplementary figure 1. Unilateral V1 lesion

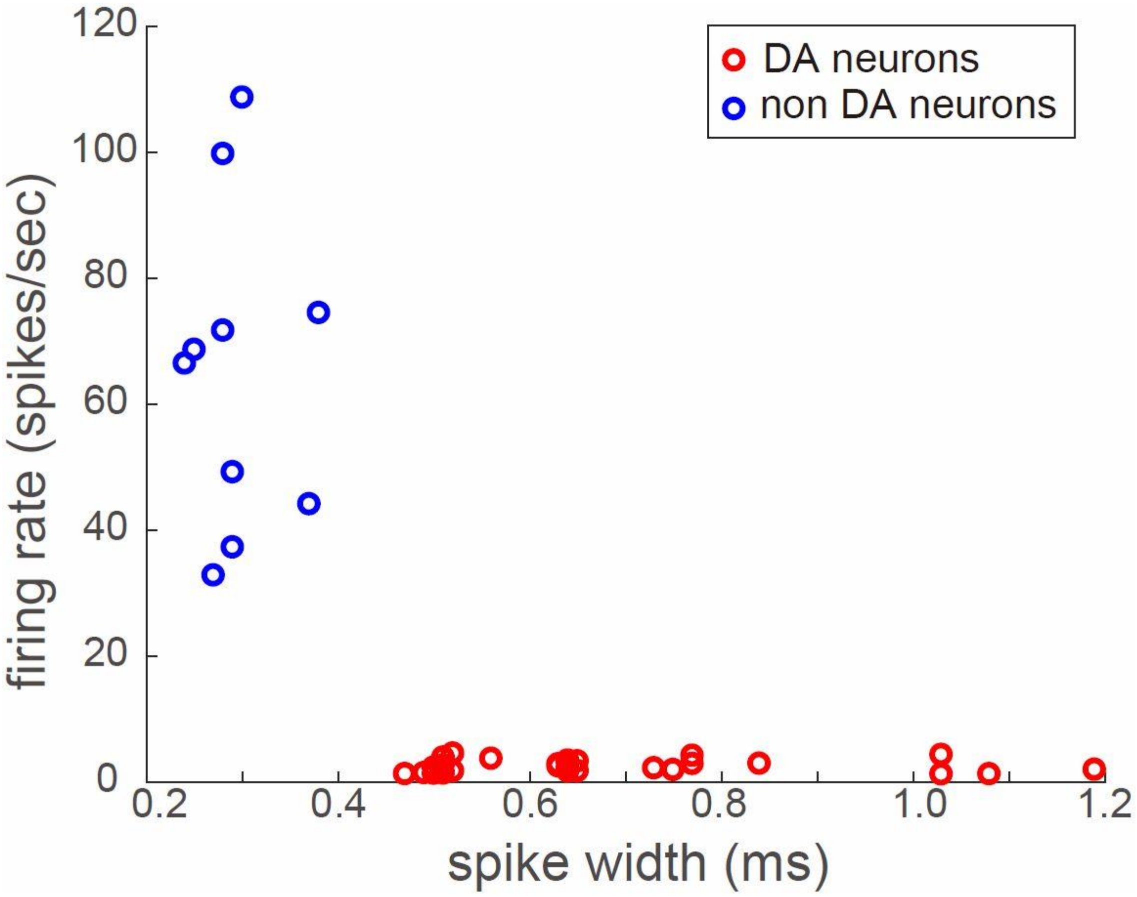

Supplementary figure 2. Criteria of our presumable DA neurons

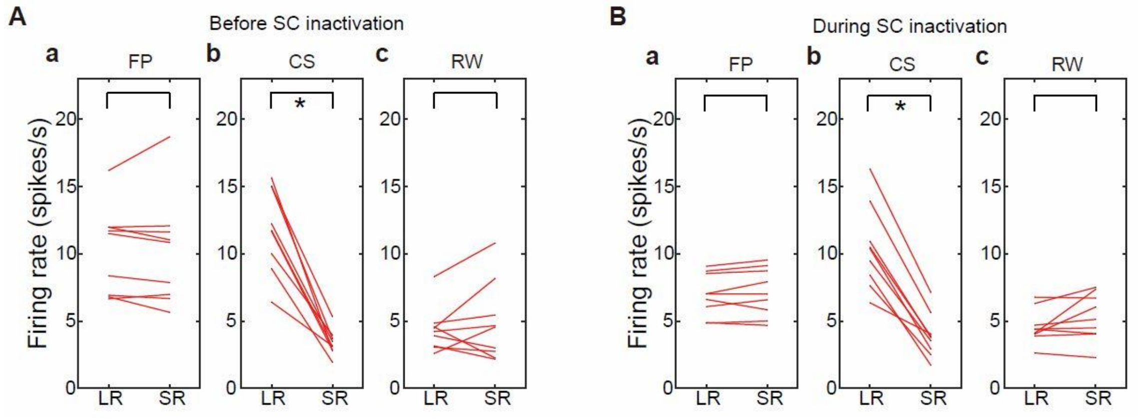

Supplementary figure 3. Comparison of firingrates between LR and SR trials before and after the SC inactivation

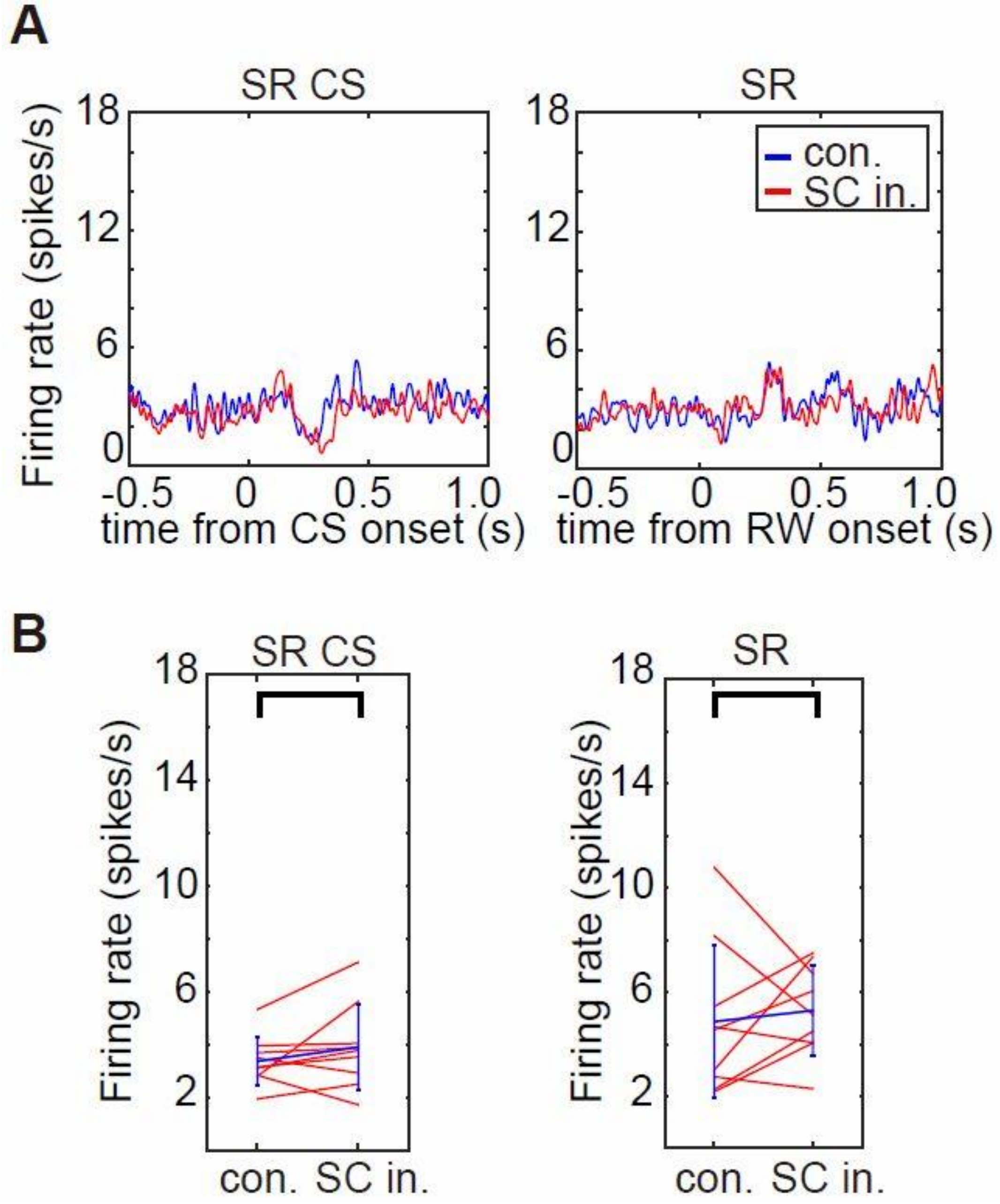

Supplementary figure 4. DA responses in SR trials during the SC inactivation

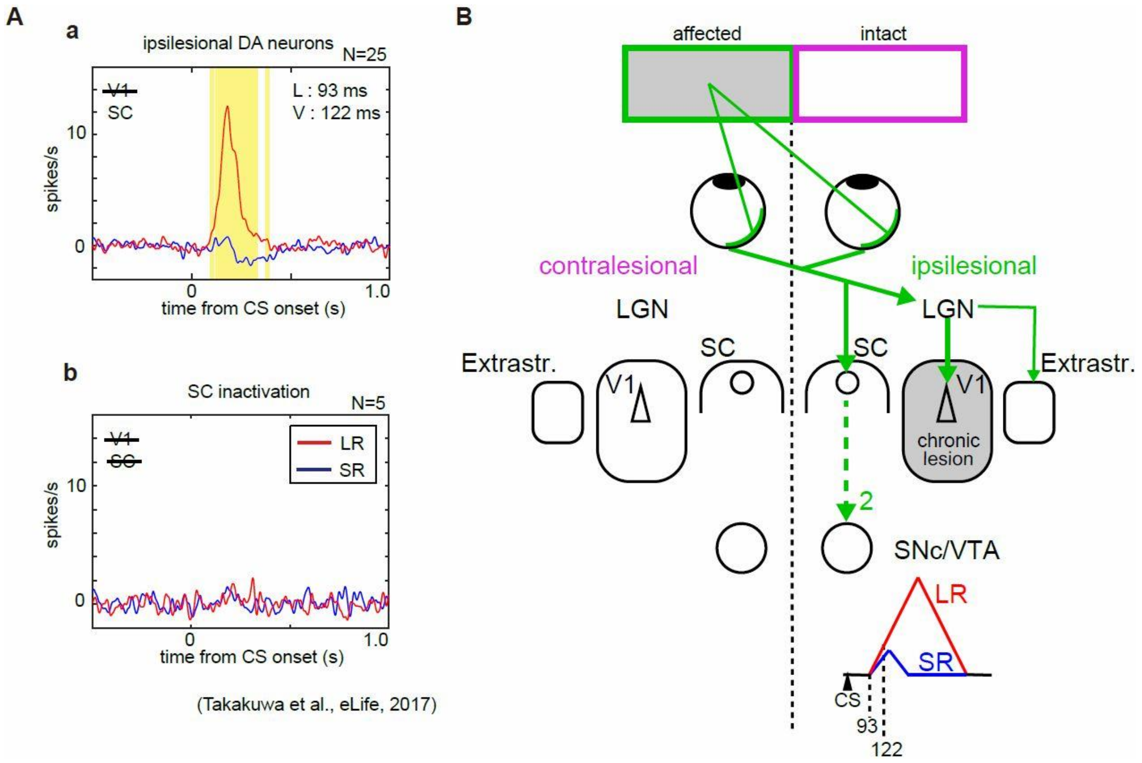

Supplementary figure 5. Visual input pathways and onsets of the DA response to visual CSs carried by the SC.

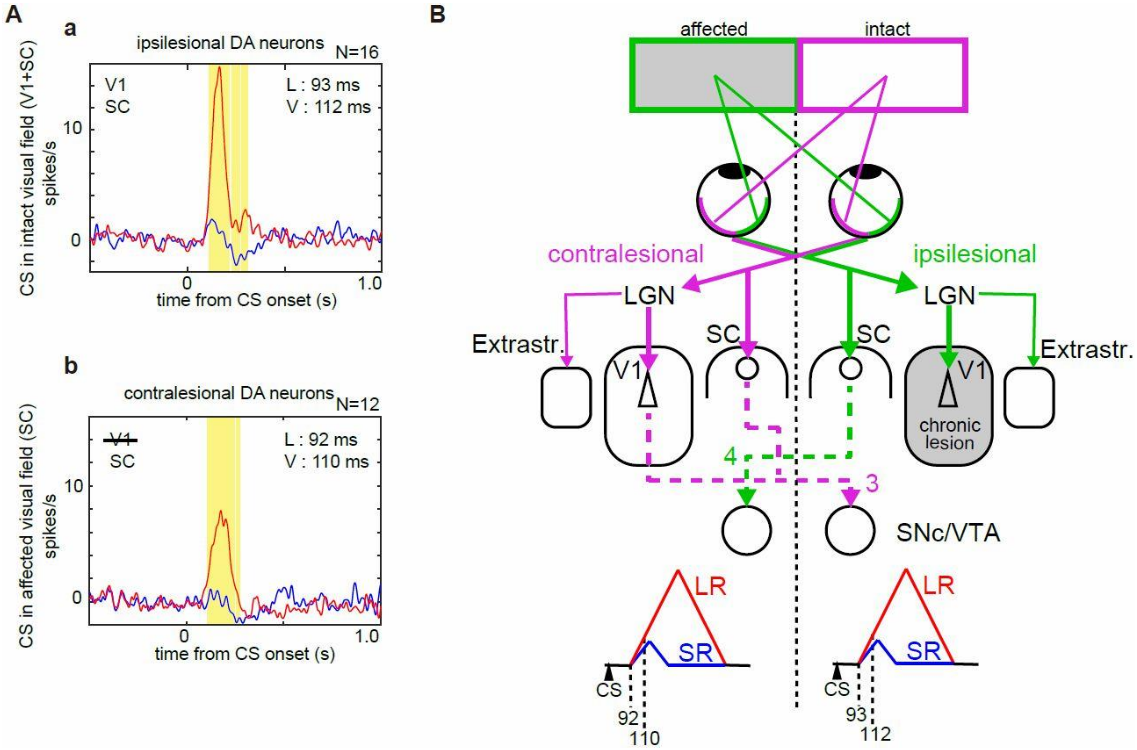

Supplementary figure 6. Visual input pathways and onsets of the DA response to visual CSs carried by both the SC and the V1

**Monkey K**					**Monkey T**			
	diff_org	CI up	CI low			diff_org	CI up	CI low
**day1**	0.236	0.925	-0.451		day1	0.207	0.534	-0.081
**day2**	0.162	0.880	-0.509		day2	0.370	0.778	-0.097
**day3**	-0.309	0.152	-0.770		day3	0.118	0.399	-0.152
**day4**	0.065	0.523	-0.403		day4	0.030	0.208	-0.154
**day5**	-0.227	0.679	-1.177		day5	0.068	0.334	-0.193
**day6**	0.264	0.932	-0.464		day6	0.041	0.226	-0.150
**day7**	0.066	0.805	-0.648		day7	0.188	0.422	-0.032
					day8	-0.056	0.129	-0.237
					day9	0.149	0.392	-0.066

Table 1. Permutation test on licking rate between before and during the SC inactivation

LR-CS				
	pre firing rate	post firing rate	CI up	CI low
**neuron 1**	12.6	8.2	8.065	0.278
**neuron 2**	12.5	11.1	4.875	-1.895
**neuron 3**	16.6	14.5	10.242	-5.909
**neuron 4**	12.2	10.0	7.037	-2.592
**neuron 5**	9.4	6.6	6.393	-1.023
**neuron 6**	15.8	12.4	11.111	-4.629
**neuron 7**	7.5	9.4	2.910	-6.183
**neuron 8**	17.0	15.6	4.993	-2.365
**neuron 9**	10.5	6.3	8.042	0.2323

SR-CS				
	pre firing rate	post firing rate	CI up	CI low
**neuron 1**	3.3	1.6	3.461	-0.230
**neuron 2**	2.8	3.5	1.377	-2.946
**neuron 3**	2.9	5.1	1.045	-5.576
**neuron 4**	3.3	2.9	2.843	-1.776
**neuron 5**	2.0	2.3	1.592	-2.389
**neuron 6**	3.3	4.1	2.985	-4.851
**neuron 7**	3.0	3.2	2.192	-2.740
**neuron 8**	6.7	6.6	2.837	-2.837
**neuron 9**	3.9	3.9	2.828	-2.828

FP				
	pre firing rate	post firing rate	CI up	CI low
**neuron 1**	11.4	6.0	7.794	3.036
**neuron 2**	11.9	9.0	4.664	1.178
**neuron 3**	11.9	7.0	8.560	1.274
**neuron 4**	6.9	6.6	2.750	-2.128
**neuron 5**	6.7	4.8	4.115	-0.398
**neuron 6**	16.1	7.0	14.071	4.020
**neuron 7**	8.3	8.7	2.950	-3.726
**neuron 8**	11.6	8.5	5.280	0.982
**neuron 9**	6.6	4.8	3.941	-0.302

**LR**				
	pre firing rate	post firing rate	CI up	CI low
**neuron 1**	3.4	3.8	1.838	-2.781
**neuron 2**	4.5	4.4	1.895	-1.625
**neuron 3**	5.1	6.3	3.786	-5.909
**neuron 4**	2.5	3.8	0.740	-3.333
**neuron 5**	3.2	2.2	3.069	-1.278
**neuron 6**	4.7	5.5	3.240	-5.092
**neuron 7**	4.6	3.9	4.001	-2.910
**neuron 8**	5.1	4.2	3.577	-1.839
**neuron 9**	8.7	6.7	5.576	-1.626

**SR**				
	pre firing rate	post firing rate	CI up	CI low
**neuron 1**	2.6	3.7	0.923	-3.230
**neuron 2**	4.3	3.8	2.209	-0.982
**neuron 3**	5.7	6.9	3.350	-5.576
**neuron 4**	4.4	6.0	1.066	-4.264
**neuron 5**	3.0	2.1	2.920	-1.327
**neuron 6**	7.3	5.5	6.573	-3.732
**neuron 7**	2.9	6.7	-0.106	-7.566
**neuron 8**	2.5	4.2	0.773	-4.384
**neuron 9**	10.5	6.5	8.722	-0.942

Table 2. Permutation test on firing rate of DA responses between before and during the SC inactivation
